# Treatment of Complex Fistula in Ano with Cable-Tie Seton: A Prospective Case Series

**DOI:** 10.5402/2011/636952

**Published:** 2011-04-28

**Authors:** Ayaz Ahmad Memon, Ghulam Murtaza, Rizwan Azami, Hasnain Zafar, Tabish Chawla, Altaf Ali Laghari

**Affiliations:** Aga Khan University Hospital, Karachi 74800, Pakistan

## Abstract

*Objective*. To determine the fecal incontinence and recurrence rate in patients with complex fistula in ano managed with cable tie seton at a tertiary care teaching hospital. *Methods*. This is a prospective case series of patients with complex anal fistula i.e. recurrent fistula or encircling >30% of external anal sphincter, managed with cable tie seton from March 2003 to March 2009. Patients were seen in the clinic after 72 hours of seton insertion under anesthesia and then every other week. Each time the cable-tie was tightened if found loose without anesthesia and incontinence was inquired according to wexner's score. *Results*. Seventy nine patients were treated during the study period with the age (mean ± standard deviation) of 41 ± 10.6 years and. The seton was tightened with a median of six times (3–15 times range). Complete healing was achieved in 11.2 ± 5.7 weeks. All the patients were followed for a minimum period of one year and none of the patients had any incontinence. Recurrence was found in 4 (5%) patients. *Conclusion*. The cable tie seton is safe, cost effective and low morbidity option for the treatment of complex fistulae-in-ano. It can, therefore, be recommended as the standard of treatment for complex fistulae-in-ano requiring the placement of a seton.

## 1. Introduction

Fistula-in-ano is one of the commonly encountered surgical problems with prevalence of 1.2 to 2.8/10,000 [1]. The classification of fistula-in-ano, as described by Parks et al. is based on the location of its tract in relation to anal sphincter muscle: intersphincteric, transsphincteric, suprasphincteric, or extrasphincteric [2]. The term complex fistula is modification of the Park's classification, which falls in any one of these conditions, that is, the track crosses >30% to 50% of the external sphincter, anterior in females, multiple tracks, recurrent, or the patient has preexisting incontinence, local irradiation, or Crohn's disease. Due to the involvement of the anal sphincter, the treatment of complex fistula poses a high risk for impairment of continence [3, 4].

Due to the lack of a single appropriate technique for the treatment of fistula-in-ano, treatment must be navigated by the surgeon's experience and judgment. The surgeon has to keep in mind the tradeoff between the extent of sphincter division, postoperative healing rate, and functional loss [3]. Whatever the type and the extent of fistula are, the principles of anal fistula surgery are to get rid of the fistula, prevent recurrence, and preserve sphincter function. Most of the fistula-in-ano has been conventionally treated by either fistulotomy, or fistulectomy, which have been proven to be effective [5]. Seton have been used to manage anal fistula from hundreds of years; however, in the literature, setons were commonly used only for high or complex anal fistula in order to avoid fecal incontinence and recurrence [6].

Seton is any string-like material which when tied through the fistula tract causes an inflammatory reaction which stimulates fibrosis that fixes and prevents retraction of the sphincter continuity when it is divided. In this way, it maintains sphincter continuity during cutting process [7]. Different types of setons are used for this purpose like silastic tube, silk, linen, braided silk, rubber band, braided polyester, vascular loop, polypropylene, nylon, cable tie, and so forth [7]. The reported incontinence and recurrence rate ranges from 0% to 62% [7] and from 0% to 16% [8], respectively, with different materials used as seton. The cable tie is very cheap, easily inserted, and provides convenient tightening in a clinic setting without need of analgesia which even can be done by the attendants if they are trained once. We have conducted this prospective study on a larger sample size to determine the incontinence and recurrence rate of cable-tie seton along with risk factors for recurrence at a tertiary care hospital.

## 2. Patients and Methods

This is a prospective data of patients with complex anal fistula, that is, recurrent fistula or encircling >30% of sphincter external anal sphincter, who were managed with cable tie seton from March 2003 and March 2009. Patients with existing preoperative incontinence, loss to followup, inflammatory bowel disease, intestinal tuberculosis, anorectal tumor, or ASA IV were excluded. 

When patients were seen in the clinic, no effort was made to define the tract or investigate the condition radiologically. They were counseled about the method of dealing with the fistula and the fact that healing and/or convalescence is likely to be prolonged. After that, ASA-I and II patients were operated as day-care cases, while ASA-III patients were operated as regular inpatients. Bowel preparation was done in all patients with clear-liquid diet 24 hours prior to surgery and/or clean enema in day care. The majority of patients preferred to have general anesthesia, albeit spinal and caudal blocks were also used at times.

**Figure 1:**
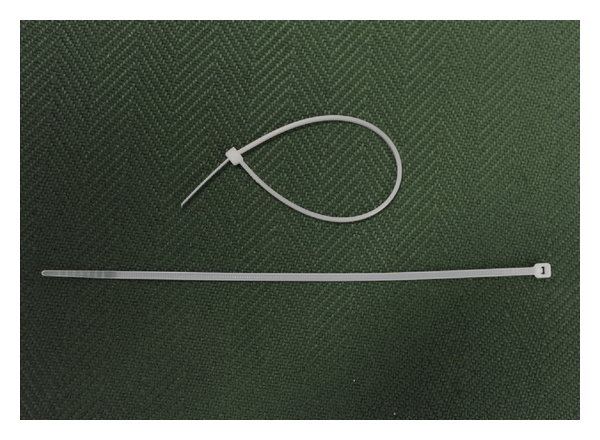
Cable-tie seton.

In the operation room, the patients were evaluated in the lithotomy position. A rigid sigmoidoscopy and proctoscopy were done prior to any intervention. Using a proctoscope, roll gauze was placed in the anal/rectal canal. An alcoholic solution of gentian violet in a 3 mL syringe was used to stain the entire tract by injecting into the external opening using the stub of a 21G needle that has been broken about 3 to 5 mm from the hub. The roll of gauze was withdrawn to identify the depth and position (circumferential) where the internal opening exists. The external opening was gently probed using a standard 3 mm blunt-tipped probe till the previously identified internal opening. The amount of sphincter superficial to the probe was evaluated. A 9- to 12-inch length of intravenous tubing was attached to the probe and drawn through the tract from the external opening to the internal opening. The area around the external opening was dissected around the tract (with the drip set tube inside it) up to the sphincter; this core of tissues was then divided and drawn off the tubing. The skin from the internal opening to the lateral portion of the tract was incised to allow the cable-tie seton (Figure 1) to settle onto the sphincter; the cable tie was then inserted into the drip set tubing from outside inwards, and the drip set tubing was pulled out from the anal side of the opening. The cable tie was then locked and tightened to make it sit loosely over the sphincter (which was then within the loop of seton).

Dry gauze dressing was used to cover the wound and retained in place using a sanitary napkin and waist thong. Paracetamol was used as the primary analgesic drug, and if not effective, then NSAID's (diclofenac) were used. Care at home consisted of hot soaks (Sitz bath)/hot sprays or washes and dry gauze cover retained by fitting undergarments. 

Patients were seen in the clinic after 72 hours to reinforce postoperative instructions and to evaluate wound. The patients were seen every other week and encouraged to walk as much as possible; at the same time, if the cable tie was tightened if found loose. On each visit, patients were asked about the fecal incontinence according to *Wexner's score* (Table 3) [9].

 The cable tie was removed by dividing it near the knuckle if the cable tie did not completely cut through despite complete healing of lateral wound or core sphincter of a centimeter diameter was left behind. 

Data was analyzed with SPSS version 17. Continuous variables were analyzed as means ± SD, where as categorical variables were analyzed as proportions and percentages. Risk factors of recurrence were analyzed with Chi square or Fisher's exact test wherever applicable.

## 3. Results

We operated on 79 patients of fistula-in-ano with cable-tie seton during March 2003 and March 2009, with age (mean ± standard deviation) of 41 ± 10.6 years. Seventy-eight (98.7%) were males, and seven (8.9%) were diabetic. Fifty-eight (73.4%) patients did not have any prior history of perianal problem, while the rest had past history of either perianal or ischiorectal abscess. Fifty-three patients (67.1%) had no previous history of surgery for fistula-in-ano, whereas rest presented with recurrent fistulae (Table 1). Fistula tract was traced and delineated in all the cases. 

The majority of patients (58.2%) had high transsphincteric fistula-in-ano and 70.9% had internal opening above dentate line. The seton was tightened with a median of six times (3–15 times range). Most of the patients tolerated the tightening session well with no or minimal analgesia, that is, paracetamol 1 gm every six hourly on as needed basis. Complete healing was achieved in 11.2 ± 5.7 weeks. All the patients were followed up for a minimum period of one year (or till healing if the period exceeded one year), and none of the patients reported incontinence. Recurrence was found in 4 (5%) patients. Although recurrence was found more in high transsphincteric fistulae, fistulae having internal opening above dentate line and external opening anterior to transverse midline (Table 2), these were statistically insignificant factors. One patient got the seton removed prematurely owing to unbearable discomfort. None of the patients had bleeding, wound infection, premature dislodgement, or slippage of the seton. 

## 4. Discussion

In this paper, we found 0% incontinence and 5% recurrence rate in 79 patients treated with cable-tie seton for complex anal fistulae. The data was collected prospectively on a large sample of patients. The data on continence was determined by validated Wexner's score in all the patients with complete followup, which includes incontinence of feces as well as flatus. All the procedures were done by a single surgeon, eliminating the bias which could have occurred with multiple surgeons. However, it is a single-arm study with no comparison group. The majority of the patients were males; this unintentional selection bias was the result of cultural norms in our country as the females prefer to be managed by female surgeons.

Different seton materials has been used with different rates of recurrence and incontinence. But whatever the material is, recurrence and incontinence rate is mainly dependent on expertise and judgment of the surgeon [7]. So, there are other factors that need to be considered during the selection of the seton. The seton should be durable, cheap (0.1 USD in our country), nontoxic/nonallergic, technically easy to tie even in clinic setting, and allows to tight repeatedly without causing pain and without anesthesia (local or general) [10, 11]. With these properties, cable tie stands out above all. It is made up of polyamide (nylon) and has got self-locking system with equally distributed clicks, which guides the operator to adequately tight it by just slipping one end without any need of further assistance/retraction. Hence, the tightening is gradual and controlled. Therefore, tightening can be easily performed in clinic setting unlike other setons, for which the patient is taken to operating room repeatedly adding into the cost and risk of analgesia to the patient. 

After tightening, none of the patients had unbearable pain for more than few minutes; this is attributed to the precise and controlled tightening achieved by cable tie as well as the fact that we did not tighten it until found loose. This controlled and gradual tightening decreased the incidence of incontinence and recurrence; however, at the cost of relatively longer time of seton in place (11.2 ± 5.7 weeks). None of the patients reported any difficulty in walking or carrying out routine activities. The cable-tie, once engaged, is self locking and is retained in place by the tissues within the loop. Free movement and abrasion/irritation of the opposing gluteal tissues was prevented by a wad of gauze on either side of the free end of the cable-tie. The wad of gauze is retained in place by an undergarment.

Cable tie has been used previously, with similar results as in our study. Gurer et al. [10] found 0% recurrence and incontinence in 17 patients treated with cable tie, with mean healing time of 38.94 days and 12% complication rate. Vatansev et al. [11] presented a series of 32 patients treated with cable tie and reported no recurrence, 15.6% incontinence rate, and mean healing time of 53 days. We conducted this study on large sample size of 79 patients and found 0% incontinence and complications with low recurrence proportion of 5%. Cable-tie seton does not suffer the problems of loosening as in elastic tie [12], cumbersome and imprecise tightening as faced in bunch of silk ties, or second procedure as in draining setons. Other techniques of treatment have been reported including fibrin glue, Ligation of Intersphincteric Fistula Tract (LIFT) and collagen plug. Metanalysis of trials on fibrin glue did not report any statistically significant difference over other techniques for recurrence or incontinence [13]; moreover, it is too expensive to be used in a low income country—the cost of fibrin glue equals the cost of entire day care procedure of seton placement. Early experience of LIFT is also promising and sounds good alternative [14]; however, besides a steep learning curve, it needs technical expertise especially for complex fistulae. 

The 0% incontinence in our series of patients can be attributed to; (1) nominal dissection and, therefore, damage to the anal sphincter muscle complex (because only the extreme lateral portion of the fistulous tract was dissected/cored out after staining the tract, probed and “intubated” with a short length of intravenous set tubing to pass the cable tie seton, (2) The sphincter muscle complex is gradually cut through because of the direct compression effect of the cable-tie and “wearing through” the tissues because of the movements produced by walking, while the depth of the tissues have a chance of adhering to each other because of the fibrosis that has occurred. Such fibrosis does not allow distraction of the sphincter muscle and the resultant incontinence.

The factors implicated in fistula recurrence include the complexity and level of the fistula, the presence or absence of a horseshoe extension, the degree of laterality of the external opening, failure by the surgeon to identify the internal opening at initial surgery, and the overall surgical experience of the operator in complicated proctologic practice [15]. In our study, we were able to identify the internal opening in all the patients without radiological investigations, and if we correlate this with the low recurrence rate, we can conclude that the most important factor is the surgeon's experience and judgment. Although this seems to be a subjective decision, but it is pragmatic and cost effective in low-income country like ours. Although the cost of MRI is more than the cost of procedure itself, with this cautious technique, we can achieve the best possible results without radiological aids. Looking into the literature, a wide range of incontinence rates is reported after cutting seton treatment, and Ritchie et al. [7] have concluded that there was no relationship between incontinence and the frequency of tightening, type of seton, or classification of fistula. Hence, we further reinforce the importance of surgeon's experience and the use of a seton having additive qualities as stated above.

## 5. Conclusion

The cable-tie seton is safe, low-cost, ubiquitous, pragmatic, precise, and a cost-effective option for the treatment of complex fistulae-in-ano. We, therefore, recommend it to treating complex fistulae-in-ano requiring the placement of a seton. It does not carry the disadvantage of repeated anesthesia and visits to the operating theater and reduces the morbidity, inconvenience, and cost to the patient.

## Figures and Tables

**Table 1:** Baseline variables.

Characteristics	Value
Age	41.3 ± 10.6
Sex	
Male	78 (98.7%)
Female	1 (1.3%)
Type of fistula	
Low transsphincteric	25 (31.6%)
High transsphincteric	46 (58.2%)
Suprasphincteric	08 (10.1%)
Internal opening	
At dentate line	23 (29.1%)
Above dentate line	56 (70.9%)
External opening from anal verge (Goodsall's rule)	
Within 2.5 cms	24 (30.4%)
Beyond 2.5 cms	55 (69.6%)
External opening according to goodsall's rule	
Anterior	44 (55.7%)
Posterior	35 (44.3%)
Outcomes	
Perfect continence	79 (100%)
Recurrence (1 year)	04 (5.1%)

**Table 2:** Risk factors for recurrence.

Characteristic	Recurrence (*n* = 4)	No recurrence (*n* = 75)	*P* value*
DM	0	7 (9.3%)	.685
Previous surgery	1 (25%)	25 (33.3%)	.60
Type of fistula			
Low	0	25 (33.3%)	
High	3 (75%)	43 (57.3%)	.29
Suprasphincteric	1 (25%)	7 (9.3%)	
Type of fistula			
Primary	3 (75%)	50 (66.6%)	
Recurrent	1 (25%)	25 (33.3%)	.31
Internal opening			
Below dentate line	0	23	
Above dentate line	4	52	.16
External opening			
Anterior	3	41	.27
Posterior	1	34	

*Chi square/Fischer's exact test.

**Table 3:** Wexner's score for fecal incontinence.

Characteristic	Never	Rarely <1/month	Sometimes >1/month <1/week	Usually >1/week <7/day	Always ≥1/day
Flatus	0	1	2	3	4
Liquid stool	0	1	2	3	4
Solid stool	0	1	2	3	4
Wears pad	0	1	2	3	4
Alteration in lifestyle	0	1	2	3	4

Range (0–20); 0 = normal continence and 20 = maximum incontinence with maximum disturbance of life style.
